# The Role of IL-1*β* in the Bone Loss during Rheumatic Diseases

**DOI:** 10.1155/2015/782382

**Published:** 2015-04-12

**Authors:** Piero Ruscitti, Paola Cipriani, Francesco Carubbi, Vasiliki Liakouli, Francesca Zazzeroni, Paola Di Benedetto, Onorina Berardicurti, Edoardo Alesse, Roberto Giacomelli

**Affiliations:** ^1^Department of Biotechnological and Applied Clinical Sciences, Rheumatology Unit, University of L'Aquila, Delta 6 Building, 67100 L'Aquila, Italy; ^2^Department of Biotechnological and Applied Clinical Sciences, University of L'Aquila, L'Aquila, Italy

## Abstract

Several inflammatory diseases have been associated with increased bone resorption and fracture rates and different studies supported the relation between inflammatory cytokines and osteoclast activity. The main factor required for osteoclast activation is the stimulation by receptor activator of nuclear factor kappa-B ligand (RANKL) expressed on osteoblasts. In this context, interleukin- (IL-) 1*β*, one of the most powerful proinflammatory cytokines, is a strong stimulator of in vitro and in vivo bone resorption via upregulation of RANKL that stimulates the osteoclastogenesis. The resulting effects lead to an imbalance in bone metabolism favouring bone resorption and osteoporosis. In this paper, we review the available literature on the role of IL-1*β* in the pathogenesis of bone loss. Furthermore, we analysed the role of IL-1*β* in bone resorption during rheumatic diseases and, when available, we reported the efficacy of anti-IL-1*β* therapy in this field.

## 1. Introduction

Bone homeostasis is maintained by a fine balance between bone resorption and bone formation. Diseases characterized by a bone loss showed a common pattern: the aberrant activation of cells responsible for bone resorption, the osteoclasts [1, 2]. Activated osteoclasts induced a “resorption lacuna,” followed by the activation of osteoblasts, which fill the lacuna producing new bone matrix. When the osteoclasts resorptive activity exceeds the function of osteoblasts to refill the resorption lacunae, the accelerated bone remodelling may result in bone loss and deterioration of cancellous and cortical bone architecture and bone fragility [3, 4]. Osteoblasts not only play a central role in bone formation by synthesizing multiple bone matrix proteins, but also regulate osteoclast maturation by soluble factors and cell-cell interaction. The main factor required for osteoclast maturation is the receptor activator of nuclear factor kappa-B ligand (RANKL), expressed on surface of osteoblasts [2]. Although osteoblasts are the major source of RANKL, this molecule may be produced by other cells such as fibroblast and T lymphocytes [5, 6].

Several inflammatory diseases have been associated with increased bone resorption and fracture rates [7, 8]. Innate and adaptive immunity cells produce inflammatory cytokines that not only perpetuate inflammation but also may activate bone degradation and inhibit bone formation. Indeed, the degree of the inflammation correlates with the extent of local and systemic bone loss [7]. In particular, different studies supported the relationship between inflammatory cytokines such as TNF-*α*, interleukin- (IL-) 6, IL-11, and IL-17, and osteoclast activity [7–9]. In this context, several published results confirm the role of IL-1*β*, to induce bone resorption and osteoporosis [10].

In this paper, we review the available literature on the role of IL-1*β* in the pathogenesis of bone loss. Furthermore, we analyzed the role of IL-1*β* in bone resorption during rheumatic diseases and, when available, we reported the efficacy of anti-IL-1*β* in this field.

## 2. IL-1***β***

The IL-1 family of ligands includes 11 members and among them IL-1*β* emerged as the primary therapeutic target for an expanding number of inflammatory conditions. The inactive IL-1*β* precursor is cleaved by caspase-1 via a protein complex called inflammasome into an active cytokine [11]. IL-1*β* binds type I (IL-1RI) and type II (IL-1RII) specific receptors. Both receptors have a single transmembrane domain and an IgG-like extracellular domain. IL-1RI has a conserved region of 212 amino acids in the cytoplasmic tail, which is known as the Toll/IL-1R domain. IL-1RII contains a signalling-incompetent cytoplasmic domain of only 29 amino acids and acts as a negative regulator of IL-1*β* signalling by serving as a docking site for IL-1*β*, thereby preventing its interaction with IL-1RI. Upon IL-1*β* binding, IL-1RI undergoes a conformational change required for the recruitment of downstream signalling molecules. Two intracellular adaptor proteins are assembled by conserved cytosolic regions called Toll- and IL-1R-like (TIR) domains: the myeloid differentiation primary response gene 88 (MYD88) and interleukin-1 receptor-activated protein kinase (IRAK) 4. Phosphorylation of IRAK4 is followed by phosphorylation of IRAK1, IRAK2, and tumor necrosis factor receptor-associated factor (TRAF) 6. TRAF6 is a ubiquitin E3 ligase that, in association with ubiquitin E2 ligase complex, attaches K63-linked polyubiquitin chains to some of IL-1 signaling intermediates, for instance, TGF-*β*-activated protein kinase (TAK-1). This mechanism facilitates the association of TAK-1 with TRAF6 and with MEKK3. These signaling pathways lead to activation of many transcription factors, such as NF-*κ*B, AP-1, c-Jun N-terminal kinase (JNK), and p38 MAPK [11, 12]. A natural IL-1 receptor antagonist (IL-1Ra) that had no agonist activity in humans is released, to limit the IL-1*β* action [12].

**Figure 1:**
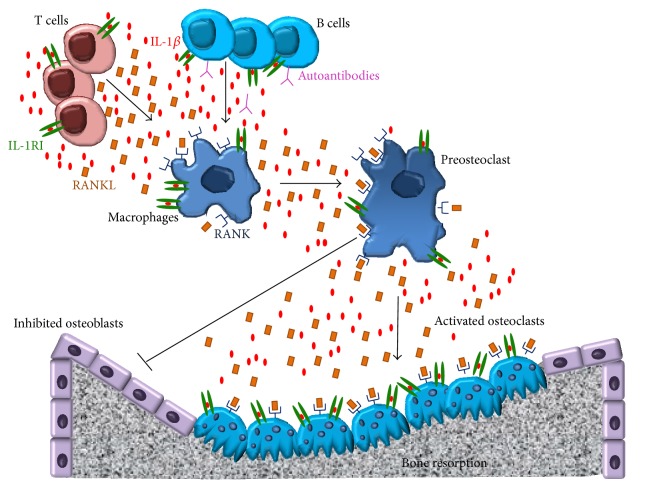
The inflammatory cascade during autoimmune and autoinflammatory diseases leading to bone resorption. Activated T cells produce IL-1*β* and RANKL. IL-1*β* stimulates T and B cells in an autocrine and paracrine fashion amplifying the inflammatory response. Macrophages after influence of IL1 and RANKL produce these 2 molecules and transdifferentiate toward preosteoclasts, which activated themselves, display strong homing to the bone, and produced higher levels of IL-1*β* and RANKL leading to the increased bone resorption.

IL-1*β* is a strong stimulator of* in vitro* and* in vivo* bone resorption [10–13]. IL-1*β* upregulates the production of RANKL enhancing its activity and stimulating osteoclastogenesis [14]. RANKL, the key osteoclastogenic cytokine, is a member of the membrane-associated TNF ligand family and it is expressed on osteoblasts, bone marrow stromal cells, activated T cells, rheumatoid synovial fibroblasts, and microvascular endothelial cells [14]. It binds the receptor activator of NF-kB (RANK), present on the osteoclast precursor cells, leading to the osteoclast differentiation and activation [15]. In particular, the RANKL-RANK interaction stimulates several transcription factors and MAP kinases, induces the expression of c-Fos family members, and promotes the nuclear translocation of both Jun proteins and NFATc1 [16]. All these effects modulate the osteoclast differentiation, activation, and survival, thus leading to the bone resorption [17]. In addition, the* in vitro* osteoclasts formation, from monocytes after RANKL- and 1,25-dihydroxyvitamin D_3_ stimulation, is mediated by the autocrine production of IL-1*β* [18, 19] (Figure 1).

IL-1*β* also regulates the production of osteoprotegerin (OPG), a natural inhibitor of RANKL. OPG inhibits osteoclast differentiation by binding RANKL [19, 20].

In addition, IL-1*β* increases prostaglandin synthesis in bone [7, 10, 13] which displays a potent resorption stimulus [10]. In fact, after inflammatory stimulus, prostaglandins, such as prostaglandin E2 (PGE2), may mediate the upregulation of RANK by activating cell-surface receptors, thus regulating osteoclast differentiation, activation, and survival [10]. Furthermore, IL-1*β* also stimulates osteoclast activity by increasing production of macrophage colony-stimulating factor (M-CSF) and inhibits osteoclast apoptosis [21, 22].

During the inflammatory osteoclastogenesis, IL-1*β* has an intimate relationship with TNF-*α* and many effects of TNF-*α*, on osteoclastogenesis, are upregulated by IL-1*β* [1, 7, 21]. In fact, experimental evidence suggests that blocking IL-1*β* and TNF-*α* results in a total arrest of bone resorption [1, 7, 21]. Furthermore, recent reports suggest that IL-1*β* strongly inhibits osteoblastogenesis, thus decreasing the new bone formation. This inhibition of osteoblasts activity is modulated via mitogen-activated protein kinase (MAPK), by activated signal transducers and activators of transcription (STATs) and by SMAD ubiquitylation regulatory factor 1 (SMURF1) and SMURF2. IL-1*β* also upregulates Dickkopf-related protein 1 (DKK1) and sclerostin (SOST) which may downregulate the osteoblasts [7].

In the last years, some specific polymorphisms of the IL-1*β* and IL-1Ra genes have been associated with an increased risk of osteoporosis. In a Chinese population of patients affected by postmenopausal osteoporosis, a strong association between the Taq I IL-1b exon 5 gene polymorphism and a reduced bone mineral density (BMD), mainly at the lumbar spine, was shown [23]. More recently, the IL-1*β* (-511C/T) polymorphism has been associated with an earlier insurgence of osteoporosis in postmenopausal women [24]. As far as the IL-1Ra gene is considered, the A1A1/A3 genotypes of the IL-1ra VNTR polymorphism were significantly more frequent in osteoporotic patients when compared with age-matched normal controls and were associated with increased risk of osteoporotic fractures [25, 26]. At present, these polymorphisms may be considered as independent risk factors for osteoporosis [24–26].

## 3. Osteoporosis

Osteoporosis is characterized by a reduction of BMD resulting from accelerated bone remodelling, which over time leads to the deterioration of trabecular architecture and loss of connectivity due to perforation of trabecular plates and rod-like structures and a decrease in mechanical strength [27]. Eighty percent of patients with osteoporosis are women, and this data is largely due to the marked loss in BMD associated with the withdrawal of estrogens, after menopause [1], which markedly increases the osteoclast activity. However, recent hypothesis suggests that the lack of estrogens may have only a minor effect [1, 27], pointing out the role of proinflammatory cytokines such as IL-1*β*, as shown in experimental models [1]. The decline in ovarian function after menopause is associated with spontaneous increases in proinflammatory cytokines, as observed in rats after ovariectomy, and these values correlated with a decrease in BMD. On the contrary, the administration of IL-1Ra, after ovariectomy, improved the BMD [28]. Mice lacking functional IL-1RI maintained their BMD after ovariectomy, while in wild-type controls the BMD was significantly reduced, further highlighting the importance of IL-1*β* in estrogen deficiency mediated bone loss [29].

In premenopausal women who underwent surgical menopause after ovariectomy, a sustained increase of spontaneous IL-1*β* secretion by peripheral blood mononuclear cells was observed and these levels were associated with a significant decrease in BMD [30]. In women who received estrogens replacement therapy after ovariectomy, IL-1*β* secretion increased transiently and returned to preoperative levels after 4 weeks of replacement therapy, confirming that the increased IL-1*β* production, associated with estrogens withdrawal, plays a major role in bone resorption [1, 30].

Forty-two early postmenopausal women received estrogen supplementation for 60 days and were further randomized in 3 arms, receiving for 3 weeks (i) anakinra, a recombinant form of human IL-1Ra, specifically designed to modify the biological response of IL-1*β*; (ii) etanercept, a recombinant form of the TNF-*α* p75 receptor, which acts as a TNF-*α* inhibitor; (iii) placebo. The assessment of serum carboxyl-terminal telopeptide of type 1 collagen (CTX) and amino-terminal telopeptide of type 1 collagen (NTX), both markers for bone resorption, and serum amino-terminal propeptide of type 1 collagen (P1NP), a marker for bone formation, was performed after 2 days of estrogen treatment and after 21 of IL-1*β* or TNF-*α* antagonism. The results showed, in both of the patients arms, a significant increase of P1NP, which correlated with the new bone formation and a decrease of serum CTX and urine NTX, thus confirming the resorptive role of inflammatory cytokines during estrogens deficiency in postmenopausal women [31] and confirming data reported in experimental models [27–29]. However, the blockade of one cytokine does not completely prevent the bone resorption observed during estrogens deficiency [31].

On the other hand, molecules that have been specifically designed to interfere with the osteoclast activity, and actually licensed for the treatment of osteoporosis, may display an anti-inflammatory effect [32]. Risedronate, a pyridinyl bisphosphonate, is a powerful inhibitor of bone resorption. It binds with high affinity to the mineralised tissue and after its deposition on the bone surface is taken up by osteoclasts during the process of bone resorption, reducing the survival and permanently disrupting the function of these cells [33]. It has been shown that, in postmenopausal women with osteoporosis, treated with oral risedronate (35 mg/week), calcium (1,000 mg/day), and vitamin D (400 IU/day) for 12 months, the serum levels of RANKL and IL-1*β* significantly decreased while no difference was found in TNF-*α* level, when compared to controls group, receiving oral calcium (1,000 mg/day) and vitamin D (400 IU/day), thus confirming the immunomodulatory effect of risedronate on improving osteoporosis, via the reduction of RANKL and IL-1*β* [33].

## 4. Rheumatoid Arthritis

Rheumatoid arthritis (RA) is a systemic autoimmune inflammatory disorder that primarily affects the synovial joints. The bone loss that occurs during RA includes three main patterns: systemic osteoporosis, juxta-articular osteopenia, and erosions [34].

The systemic rheumatoid inflammatory process is strongly linked to systemic osteoporosis. Higher levels of proinflammatory cytokines observed during the rheumatoid process lead to an increase of osteoclast differentiation and activation [35]. In addition, the degree of bone damage correlates with the number of macrophages infiltrating the synovial pannus-bone interface, which are the primary source of inflammatory cytokines such as IL-1*β*, IL-6, and TNF-*α*. In this context, IL-1*β* acts directly modulating the expression of osteoclast differentiation factors such as RANKL [36, 37]. Furthermore, IL-1*β* may also act on osteoclast progenitors, stimulating osteoclastogenesis. IL-1*β* released by macrophages and T cells seems to regulate the differentiation toward osteoclasts, thus increasing their activity [34]. On the other hand, several anti-inflammatory cytokines, such as IL-4, IL-13, and IL-18 that inhibit osteoclastogenesis, were shown to be decreased in RA [34]. As far as the role of transforming growth factor- (TGF-) *β* is concerned, conflicting results are available in literature, and both inhibition and induction of osteoclastogenesis have been reported [34, 38]. In fact, it has been reported that TGF-*β* may inhibit resorption in organ culture and osteoclast-like cell formation in bone marrow cultures [34]. Recently, a study showed that TGF-*β* might upregulate the expression of osteoclastogenesis-related genes in mouse bone marrow macrophages [38].

These findings support the clinical evidence of a higher fracture risk in RA patients. In addition, several studies indicate that this risk increases approximately 1.5- to 2-fold [38–40]. Premature bone loss may occur in young RA male patients in which a reduction in new bone formation is evident when RA patients are compared with age- and gender-matched controls [39].

The juxta-articular osteopenia occurs in that portion of bone, in the proximity to the inflamed joints which are exposed to high levels of proinflammatory cytokines produced locally by the synovium in the affected joints. Focal erosions occur at the edge of the articular surface where the proliferating pannus invades the subchondral bone. In different experimental models of arthritis, it has been shown that an increased production of IL-1*β* is able to directly activate osteoclasts, thus inducing the bone erosions [34, 41]. In addition, IL-1*β* in association with other inflammatory cytokines such as TNF-*α* and IL-6 may amplify these effects inducing bone loss via the modulation of the synovial hypertrophy, the leucocyte infiltration, and the pannus formation [41]. Furthermore, the deletion of the gene encoding for IL-1Ra was associated with the occurrence of spontaneous polyarthropathy with bone erosions [42]. IL-1*β* plays an important role in the degradation of articular cartilage by stimulating both synovial fibroblasts and chondrocytes to secrete matrix metalloproteinases (MMPs), cathepsins, and mast cell proteinases. In fact, it has been shown that IL-1*β* upregulates the MMPs expression via a complex transcriptional regulation, which degrade cartilage by hydrolysing matrix constituents [43]. In rheumatoid synovium, the upregulation of IL-1*β* increases MMPs, which in turn aggravates synovial inflammation, increased joint destruction, and bone resorption [34, 43]. Furthermore, this upregulation supports the bone resorption, increasing the RANKL expression in cultured synovial fibroblasts and in the T cells from RA patients [44–46]. Finally, IL-1*β* impairs the synthesis of new matrix components in RA patients [34, 43].

In different animal models of arthritis, the antagonism of IL-1*β* significantly reduced cartilage damage, independently of a decrease of the inflammatory markers. On the contrary, TNF-*α* blockers were able to strongly decrease the inflammation but did not prevent cartilage damage [47]. Furthermore, in adjuvant arthritis models, the antagonism of IL-1*β* with neutralizing antibodies decreased both the disease activity and the bone resorption [48, 49]. Similarly, transferring the IL-1Ra gene in a chronic relapsing streptococcal cell wall-induced arthritis experimental model, a decrease of inflammatory markers as well as a reduction in cartilage and bone erosions was described [50]. Similarly, intra-articular transfer of the human IL-1Ra gene in an antigen-induced arthritis experimental model decreased the cartilage breakdown and preserved cartilage matrix synthesis [51]. Moreover, in severe combined immunodeficiency (SCID) mice, implanted with RA synovium and normal human cartilage, the transfer of the human IL-1Ra gene into the synovial fibroblasts prevented the chondrocyte-mediated cartilage degradation [52].

Taken together, these findings suggest that blocking IL-1*β* may have a possible therapeutic benefit on bone and cartilage destruction in RA. Different studies with anakinra have been performed to evaluate its protective effect on both cartilage and bone [53, 54]. In a study employing the Larsen radiographic scores for outcome of RA patients, a significant improvement was reported with anakinra treatment when compared with placebo after 24 weeks [53]. Another study evaluating 419 patients with active RA showed that the radiographic progression, assessed by Larsen score and by Genant score, was significantly delayed in anakinra group [54]. Furthermore, in a systematic review including 5 trials involving 2876 patients, 781 randomized to placebo and 2065 to anakinra were analyzed. The results showed a significant improvement in Larsen radiographic scores in anakinra treated patients groups when compared with placebo groups [55]. Finally, anakinra was able to reduce the daily intake of glucocorticoids in RA patients in many clinical trials [53–55]. This is considered a significant added benefit when considering the harmful effect of steroids on bone metabolism.

## 5. Spondyloarthritides

The spondyloarthritides include ankylosing spondylitis (AS), reactive arthritis, psoriatic arthritis (PsA), inflammatory bowel disease-associated spondyloarthropathy, and undifferentiated spondyloarthropathy [56]. AS is one of spondyloarthritides and it is an inflammatory disease involving primarily the axial skeleton and sacroiliac joints [56].

AS patients classically show an association of new bone formation, the syndesmophytes, and increased bone resorption [57]. In these patients, the occurrence of both osteoporosis and vertebral fractures was increased when compared with healthy controls [58–61]. The etiology of osteoporosis in AS patients may be linked to inflammatory process and cytokines productions. In fact, increased levels of inflammatory cytokines, associated with decreased levels of OPG, have been detected in AS patients with osteopenia [62, 63]. Bone samples, obtained from zygapophyseal joints of AS patients, revealed increased osteoclast activity and strong infiltration of T and B cells [64].

AS is characterized by paradoxical and simultaneous bone destruction and formation, occurring in proximal anatomical sites [65]. The key role of inflammatory cytokines such as TNF-*α* in AS pathophysiology has been strongly confirmed by the success of anti-TNF-*α* therapy in these patients [66]. In fact, TNF-*α* blockers reduce signs and symptoms of AS, which results in better physical function and quality of life [65, 66]. However, little or no effect on structural remodelling is achieved [67–69]. Only few data explored the efficacy of anti-IL-1*β* in the treatment of AS [70]. In one study, anakinra was not able to induce any improvement of sign and symptoms of AS, and unfortunately this study did not evaluate the bone loss or new formation [71].

## 6. Other Autoimmune Diseases

Many studies reported an increased occurrence of both osteoporosis and higher prevalence of vertebral and peripheral fractures in patients affected by systemic lupus erythematosus (SLE). The bone loss in SLE is most prominent in the lumbar spine and may be already present at diagnosis [72, 73]. Patients with SLE have a significantly higher risk of vertebral fractures, the majority of these fractures occurring in premenopausal women [74, 75], and the presence of the fractures was independent of BMD as well as of the glucocorticoids treatment. IL-1*β* and other proinflammatory cytokines are increased in patients with SLE and they may further contribute to the development of osteoporosis [21]. The chronic SLE inflammation induces RANKL production from activated T cell supporting osteoclastogenesis and inhibiting osteoblastogenesis [75, 76].

Several studies showed higher prevalence of osteoporosis in systemic sclerosis (SSc), whereas the BMD values were significantly lower, compared to the general population, mirroring what was already observed in patients with RA [77, 78]. Both the chronic inflammation and IL-1*β* activity were found to correlate with the reduction of BMD in SSc patients [72, 78].

Furthermore, an increased risk of bone loss and osteoporosis in adult patients affected by dermatomyositis/polymyositis and in patients affected by Behcet's diseases has been demonstrated which appears to be linked to inflammatory processes and production of IL-1*β* [79, 80].

Although available literature showed conflicting results about the use of anti-IL-1*β* in these diseases [7, 11, 12, 21, 80], further studies are needed to elucidate whether this drug may be useful in the treatment of bone loss during autoimmune diseases.

## 7. Chronic Nonbacterial Osteomyelitis

Autoinflammatory bone diseases are caused by seemingly unprovoked activation of the innate immune system leading to an inflammatory process of the bone. The IL-1*β* pathway dysregulation seems to be implicate in the pathogenesis of this condition [81]. The bone lesions are characterized by chronic inflammation without any evidence of infection [82, 83]. The most common autoinflammatory bone disease is chronic nonbacterial osteomyelitis (CNO) [81]. CNO is a systemic disease that can also affect the skin, joints, gastrointestinal tract, and lungs [81]. Although radiographically bone lesions are suggestive of infectious osteomyelitis, biopsies are typically sterile and the clinical picture of the disease improves with the use of anti-inflammatory medications. Bone lesions tend to cluster around the metaphysis and the most common CNO sites are the femur, tibia, pelvis, calcaneus, ankle, vertebrae, and clavicle [81–83].

The chronic inflammation during CNO seems to be caused by activation in the innate immune system [84]. In particular, after activation by the inflammasome, caspase-1 processes IL-1*β*, removing the amino-terminal amino acids to release mature, active IL-1*β* [84]. Furthermore, there is evidence that reduction of IL-10 inhibitory pathway might be linked to the development of CNO. In fact, experimental evidence showed that lipopolysaccharide- (LPS-) stimulated CNO monocytes have a decreased production of IL-10 [85].

NSAIDs are the gold standard therapy for CNO [81]. Naproxen was demonstrated to induce a complete response in the majority of patients [86]. Indomethacin might be more effective than naproxen but is associated with more side effects [87]. Single case studies and small case series have been published, addressing the treatment of CNO with various medications, including corticosteroids and disease-modifying antirheumatic drugs such as methotrexate and sulfasalazine, with positive results [88]. TNF-*α* antagonist treatment has been used in CNO, showing encouraging results [89, 90]. Recently, the antiresorptive aminobisphosphonate pamidronate has also been used for the treatment of CNO. This drug, inactivating osteoclasts and displaying an anti-inflammatory mechanism, seems able to decrease pain and modify the clinical course of the disease [91, 92]. Other biologic medications have been used, including anakinra, which showed the ability to improve the bone manifestations of the disease [91]. In a case report, a 6-year-old female presented a 3-month history of bony pain affecting ankles, ribs, and clavicles. Bone biopsy revealed mixed inflammatory cell infiltrate and no infection or malignancy, thus confirming CNO. After starting treatment with high dose of steroids and pamidronate, which did not reach any success, anakinra was added. After 6 weeks of therapy, the clinical picture significantly improved as well as the parameters of disease activity [93].

## 8. Other Autoinflammatory Bone Diseases

Autoinflammatory bone diseases include SAPHO (synovitis, acne, pustulosis, hyperostosis, and osteitis) syndrome, Majeed syndrome, deficiency of interleukin-1 receptor antagonist (DIRA), and cherubism. These disorders are characterised by activation of innate immune system, with recurrent inflammatory flares, linked to dysregulation of the IL-1*β* pathway, resulting in sterile bone inflammation [94, 95].

During autoinflammatory bone diseases, IL-1*β* is processed and activated via nucleotide-binding domain and leucine-rich repeat containing family pyrin (NLRP3) inflammasome, that is, large multimeric procaspase-1 activating platforms. This inflammasome is composed of a sensor protein, that is, NLPR3, an adapter protein that is apoptosis-associated speck-like protein containing a CARD (ASC), and a caspase-1. Activation of inflammasomal sensor triggers the cleavage of procaspase-1 to active caspase-1, which processes pro-IL-1*β* to mature IL-1*β*. Autoinflammatory bone diseases may be associated with bacterial trigger such as* Propionibacterium acnes* as well as genetic susceptibility or gene mutations such as the mutations in Pstpip2, LPIN2, and IL1RN, which promote the aberrant activation of the innate immune system leading to dysregulation of the IL-1*β* pathway and resulting in bone inflammation [81, 94, 95].

SAPHO syndrome is an autoinflammatory disease that affects both skin and bones, which has been diagnosed primarily in adults [96]. The clinical manifestations of SAPHO include chronic recurrent multifocal osteomyelitis, palmoplantar pustulosis, severe acne, and psoriasis with neutrophilic predominance [97]. The first-line treatment of SAPHO is typically NSAIDs [98]. Other agents that have been used are methotrexate, oral corticosteroids, colchicine, and sulfasalazine [81]. TNF-*α* antagonists were used for SAPHO syndrome with a beneficial response [90]. Bisphosphonates have been used with a clinical improvement [81].

Majeed syndrome is a rare autosomal recessive disorder that is characterized by a clinical triad of features: chronic recurrent multifocal osteomyelitis, congenital dyserythropoietic anemia, and inflammatory dermatosis [99]. This disease is associated with recurrent fevers, severe pain, chronic anemia, and soft tissue swelling that typically affects large joints. Typically, NSAIDs and oral corticosteroids have been used with variable success [100]. Recently, the efficacy of anti-IL-1*β* treatment was shown. Two patients, who failed treatment with a TNF-*α* inhibitor, were treated with anti-IL-1*β* antibody with dramatic clinical, laboratory, and radiologic improvement of the bone damages [101].

DIRA is an autosomal recessive autoinflammatory disorder and it is caused by a mutation ILIRN gene [102]. DIRA is potentially life threatening and may mimic neonatal sepsis. An early correct diagnosis is a critical point to prevent multisystem organ damage and death [103]. The characteristic clinical presentation includes generalized pustulosis, osteitis, periostitis, and systemic inflammation. Within the first few weeks after birth, pustular rash and systemic signs of inflammation develop. In DIRA, the osteitis is severe, with extensive bone involvement, a multifocal osteolytic pattern of disease, and marked periostitis [104]. These bony lesions typically affect long bones and vertebral bodies and have a predilection for the proximal femur. Vertebral involvement and morbidities are common. Collapse of the vertebrae caused by osteolytic lesions can occur and cause cervical vertebral fusion. The bone biopsy in DIRA is characterized by purulent nonbacterial osteomyelitis, associated with fibrosis and sclerosis of the affected bones [103]. The genetic cause of this disease suggested the usefulness of IL-1*β* antagonism as treatment of DIRA. In fact, in these patients, anakinra improved promptly skin findings and the bony lesions. Furthermore, the systemic inflammation may be resolved by a long-standing therapy with IL-1*β* antagonism [105, 106].

Cherubism is an autosomal-dominant autoinflammatory bone disorder affecting the maxilla and mandible [107]. The bony changes of the jaw give these children's faces a chubby-cheeked appearance; hence, the disorder was named after their likeness to cherubs depicted in Renaissance art. Cherubism is driven by 2 mechanisms: on one hand, the macrophage activity leading to high levels of IL-1*β* and TNF-*α* with subsequent inflammation and, on the other hand, osteoclast activation causing excessive bone resorption, independent of the inflammatory status [108]. No specific therapies have been identified for cherubism. Recently, patients were unsuccessfully treated with adalimumab, an anti TNF-*α* monoclonal antibody [109, 110].

On the other hand, blocking IL-1*β* by anakinra in patients with the autoinflammatory syndrome neonatal-onset multisystem inflammatory disease (NOMID), a disease caused by autosomal dominant mutations in CIAS1 or NLRP3 [111], reduces systemic and organ-specific inflammation but not the bone manifestations. A study that enrolled 26 NOMID patients treated with anakinra for at least 36 months showed a significant improvement in clinical outcome at 36 and 60 months; central nervous system inflammation was suppressed, as shown by the decrease in white blood cell counts from cerebrospinal fluid, albumin levels, and opening pressures. On the contrary, the bony lesions progressed during the treatment period. Bony overgrowth was present in 10 out of 26 patients at baseline; some patients displayed joint contractures and limb length discrepancy. Despite anakinra therapy, the volume of the bony lesions increased significantly. In patients with unilateral bone lesions, no new bone lesions developed in patients while they were receiving anakinra therapy. The results of this study showed that although anakinra is well tolerated and provides sustained efficacy in the treatment of NOMID, bony lesions, in these patients, did not improve after IL-1*β* blockade [112].

## 9. Conclusion

During autoimmune and autoinflammatory bone diseases, although different therapeutic interventions may decrease the rate of inflammation, as well as the clinical picture of the diseases, a continuous bone loss may be observed. Furthermore, the bone loss associated with chronic inflammation represents a significant and often insufficiently recognized medical need. Of interest, the IL-1*β* antagonism has been shown to reduce bone loss during RA, a prototypic autoimmune, systemic, and inflammatory disease. In the future, a better knowledge of the interplay between inflammatory molecules and infiltrating cells might lead to a significant improvement in the therapeutic approach of the bone loss, which is associated with these conditions.
